# Circadian rhythms of microbial communities and their role in regulating nitrogen and phosphorus cycling in the rhizosphere of tea plants

**DOI:** 10.1093/hr/uhae267

**Published:** 2024-10-09

**Authors:** Miao Liu, Junhua Wang, Zhengzhen Li, Xin Li, Helena Korpelainen, Chunyang Li

**Affiliations:** College of Agriculture and Biotechnology, Zhejiang University, Hangzhou 310058, China; College of Life and Environmental Sciences, Hangzhou Normal University, Hangzhou 311121, China; College of Life and Environmental Sciences, Hangzhou Normal University, Hangzhou 311121, China; Key Laboratory of Tea Quality and Safety Control, Ministry of Agriculture and Rural Affairs, Tea Research Institute, Chinese Academy of Agricultural Sciences, Hangzhou 310008, China; Key Laboratory of Tea Quality and Safety Control, Ministry of Agriculture and Rural Affairs, Tea Research Institute, Chinese Academy of Agricultural Sciences, Hangzhou 310008, China; Department of Agricultural Sciences, Viikki Plant Science Centre, University of Helsinki, P.O. Box 27, FI-00014 Helsinki, Finland; College of Agriculture and Biotechnology, Zhejiang University, Hangzhou 310058, China

## Abstract

The circadian clock mediates metabolic functions of plants and rhythmically shapes structure and function of microbial communities in the rhizosphere. However, it is unclear how the circadian rhythm of plant hosts regulates changes in rhizosphere bacterial and fungal communities and nutrient cycles. In the present study, we measured diel changes in the rhizosphere of bacterial and fungal communities, and in nitrogen (N) and phosphorus (P) cycling in 20-year-old tea plantations. The fungal communities were more stable in their responses to circadian changes than bacterial communities in the rhizosphere of the cultivars LJ43 and ZC108. Nevertheless, fungal genera with circadian rhythms were more numerous and had a higher abundance at midnight. Organic P and N mineralization in the rhizosphere was more intensive in LJ43 under day–night alterations, while inorganic N and P cycling was more easily affected by circadian rhythms in ZC108. The rhizosphere denitrification encoded by the genes *AOA* and *AOB* was intensive in the morning, irrespective of tea cultivar. Genes related to rhizosphere N fixation (*nifH*) and denitrification (*nosZ* and *nirK*) expressed at greater levels in ZC108, and they reached a peak at midnight. Moreover, the diel rhythm of rhizosphere microbial communities in ZC108 largely regulated dial changes in N and P cycling. These results suggested that the bacterial and fungal communities in the rhizosphere respond differently to circadian rhythms, and they vary between tea cultivars. The timing of bacterial and fungal cycling largely regulates rhizosphere N and P cycling and their ecological functions.

## Introduction

Rhizosphere microorganisms, sometimes considered as the second genome of plants, can form functional symbiotic holobionts with plants. Plant hosts show strong selection for their rhizosphere microbiomes via nutrient exchange between the roots and soil, which is largely dependent on the responses of plants to environmental signals, such as temperature and light [1, 2]. The circadian clock of plants is synchronized with temperature and light–dark cycles, influenced by diverse processes, and connected to the structure and function of microbial communities in the rhizosphere [3, 4]. A misfunction of the circadian clocks in the plant host causes changes in the rhizosphere microbiome, affecting plant production and health [5, 6].

Most microbiomes have short reproductive times and possess circadian clocks [7]. Even so, many evidence suggested the existence of circadian clocks and rhythmicity in prokaryotes and fungi [8, 9]. Some fungi exhibit circadian rhythmicity through their inherent core oscillatory components (*etc.* Neurospora crass) or physiological rhythmicity, including spore development and discharge [10]. Some non-photosynthetic bacteria may develop inherent rhythmicity because of zeitgeber cycles [7, 11]. Additionally, the plant circadian clocks influence the composition and transcriptomic activity of the rhizosphere microbiome, such as *Brachypodium distachyon*, *Arabidopsis thaliana*, and *Hordeum vulgare* [2, 5]. For example, diurnal changes were observed in samples of *A. thaliana*, and ~10% of the bacterial taxa showed rhythmic changes in relative abundance [2]. The loss of function of plant circadian clock genes influences the assembly and abundance of rhizosphere bacterial and fungal communities [2, 5].

The plant circadian clock causes oscillations in the availability of carbon (C), water, or nutrients, which drive diurnal changes in the rhizosphere microbiome [2, 12]. Earlier studies found that concentrations of certain exudates vary over the course of the day [13, 14]. For example, higher rates of mucilage exudation were detected at night but flavonoids and catechin were prevalent during the day [15, 16]. Rhizosphere water is also diurnally depleted, which causes diurnal changes in the nutrient flow in the rhizosphere zone [17]. Such rhythmic change in rhizosphere resource availability may cause diurnal changes in microbial structure and abundance [18, 19]. The rhythmicity of plant roots, rhizosphere pH, and defense signaling molecules may also influence diurnal changes in microbial composition and abundance [20–22]. The plants’ responses to daily changes largely depend on the plant species, genotype, and developmental time [5, 23], which consequently leads to microbial diurnal fluctuations via daily changes in plant functional traits, root exudates, and responses to the environment. Tea plants have different genetic diversities and show different morphological, physiological, and molecular traits as well as responses to environmental factors [24, 25]. However, the effect of how environmental alterations on the circadian clocks of bacterial and fungal communities in the rhizospheres of different tea cultivars is not clear.

Circadian clocks regulate the physiological and behavioral processes of organisms to synchronize them with environmental alterations, which allows reduced temporal competition among sympatric species and promotes their survival in rhythmic systems [3, 26]. Soil microbes regulate nutrient cycling and form relationships with plants through their roots. Differences in microbial structure affect functional diversity, such as nutrient transformations and plant health [2, 27]. Rhizosphere microbes may affect plant growth by regulating the availability of soil nutrients and increasing access to soil nutrients, thus contributing to plant productivity and health [28, 29]. Circadian rhythms of microbial communities in the rhizosphere may change the environment and synchronize physiological processes in the rhizosphere. For example, the rhizosphere microbiome can modulate flowering time by producing auxin and influencing N availability [30]. Furthermore, core microbial taxa with circadian rhythms in the rhizosphere are involved in C, N, P, and energy metabolism [31]. Microbial biogeochemical cycling processes, such as N_2_O emission, were also reported to exhibit diel rhythmicity [32]. Loss of function of genes related to circadian clocks in *Medicago truncatula* was found to reduce root nodulation and symbiotic N fixation, suggesting a circadian influence on beneficial microbial interactions and ecological functions [6]. The composition of microbial communities in the rhizosphere is predominantly determined by soil properties and environmental factors but is also strongly affected by the genotype of the plant host [33–35]. However, the effects of tea cultivar on the daily rhythmicity of the rhizosphere nutrient cycling process are largely unknown.

Tea (*Camellia sinensis* L.) is a perennial evergreen cash crop and widely distributed in tropical and subtropical regions. ‘Longjing43’ (LJ43) and ‘Zhongcha108’ (ZC108) are widely cultivated in China, where the area of tea cultivation has recently continued to expand due to the high economic value of tea. ZC108 was generated from the LJ43 cultivar via irradiation, and had a higher leaf N content than that in LJ43 [36]. ZC108 is more resistant to anthracnose than LJ43 as evidenced by the induced hypersensitive response, hydrogen peroxide (H_2_O_2_) accumulation, and upregulated expression of genes related to H_2_O_2_ production, secondary metabolism, and cell death [24]. Contrastingly, LJ 43 has a higher photosynthetic capacity, soluble sugar polyphenols accumulation in leaves, and showed higher N uptake ability than that of ZC108 [25, 37]. Such physiological and molecular differences in LJ43 between ZC108 affect the abundance and diversity of bacterial and fungal communities [38, 39]. However, the circadian rhythms of the rhizosphere bacterial and fungal communities in ZC108 and LJ43 and their effects on nutrient cycling and microbial ecological functions remain unknown. Therefore, we attempted to answer the following questions: (1) Do bacterial and fungal communities change rhythmically during the day–night cycling? (2) How do tea cultivar affect the circadian rhythms of bacterial and fungal components? (3) Do rhythms affect nutrient processes and ecological functions in the rhizosphere? We chose ZC108 and LJ43 in 20-year-old tea plantations and these aged tea plants had stable production and tea quality. Understanding the rhizosphere microbial structure and functions provides theoretical support for the sustainable management of tea plantations.

## Results

### The composition and distribution pattern of dominant bacterial and fungal taxa

**Figure 1:**
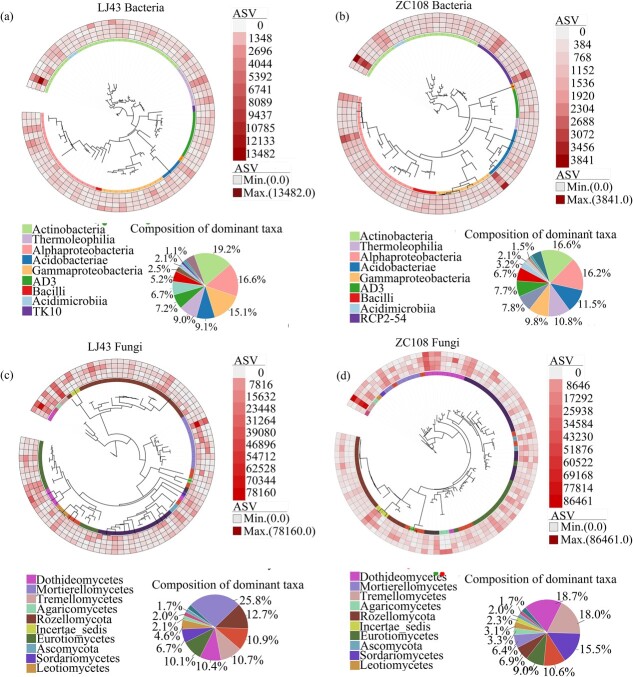
Phylogenetic relationships, taxonomic composition, and differential ASVs in the rhizosphere of ‘Longjing43’ (LJ43) (*n* = 78 and 94 for bacteria and fungi, respectively) (a, c) and ‘Zhongcha108’ (ZC108) (*n* = 82 and 96 for bacteria and fungi, respectively) (b, d). The phylogenetic tree was constructed using taxa with a relative abundance of >0.5%.

We identified the dominant bacterial and fungal taxa with a relative abundance >0.15% among all sequences in each tea cultivar (effective taxa with presence in >60% of all samples) (Fig. 1a–d). The dominant bacterial amplicon sequence variants (ASVs) were mainly affiliated with Actinobacteria, and fungal ASVs mainly belonged to the Rozellomycota class, with a relative abundance of 20%–27% within each tea cultivar. The composition of dominant bacterial taxa showed a similar pattern in the two tea cultivars: there were two dominant taxa with a proportion >10%, namely Actinobacteria and Alphaproteobacteria (Fig. 1a, b). However, the dominant fungal classes in the rhizosphere had different patterns in LJ43 and ZC108 (Fig. 1c, d). More than 10% of the fungal taxa were affiliated with Mortierellomycetes, Rozellomycota, Tremellomycetes, Dothideomycetes, and Eurotiomycetes in LJ43, but with Dothideomycetes, Tremellomycetes, and Sordariomycetes in ZC108 (Fig. 1c, d). The variation in the abundance of dominant fungal taxa between midnight (24:00) and midday (12:00) was more dynamic than that of bacteria (*c.* >25% and 50% differential taxa in LJ43 and ZC108, respectively). About 13% and 24% of bacterial taxa were upregulated, and 7% and 8% of bacterial taxa were downregulated from midnight to midday in the rhizosphere of LJ43 and ZC108, respectively (Fig. 1a, b). Contrarily, in fungi, the upregulated taxa from midnight to midday accounted for 38% and 26%, and the downregulated taxa for 17% and 26% in the rhizosphere of LJ43 and ZC108, respectively (Fig. 1c, d).

### The composition of microbial communities in the rhizosphere

**Figure 2:**
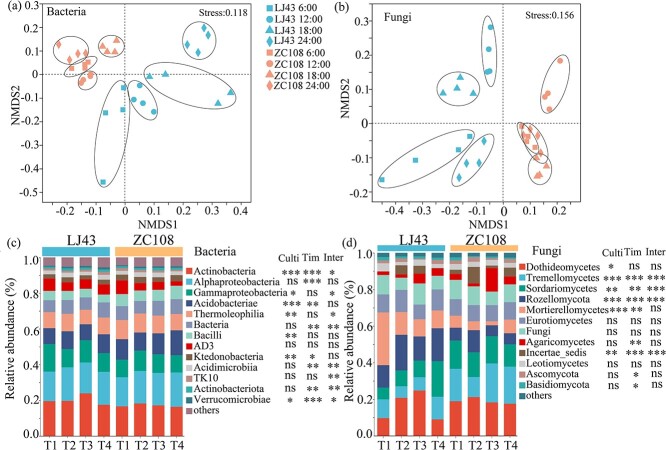
Non-metric multi-dimensional scaling (NMDS) based on Bray–Curtis dissimilarity matrices of bacterial (a) and fungal (b) communities including all samples, the dominant classes with a minimum relative abundance of 1% and the multiple comparisons of the dominant bacterial (c) and fungal classes (d) in the rhizosphere of ‘Longjing43’ (LJ43) and ‘Zhongcha108’ (ZC108). Asterisks in rectangular boxes mean significant differences in the relative abundance between cultivars (culti), among times (tim), and their interaction (inter). Values are expressed as means ± SE (*n* = 4). The significance values of variances are shown as follows: ^*^0.01 < *P* ≤ 0.05; ^**^0.001 < *P* ≤ 0.01; ^***^P ≤ 0.001; *ns*, not significant.

The bacterial and fungal community index (Shannon) was not affected by the tea cultivar (Fig. S1). Diurnal cycling did not affect fungal diversity, but it regulated bacterial community diversity, irrespective of tea cultivar (Fig. S1). Significant dissimilarities were detected between tea cultivars and diurnal cycling (Fig. 2a, b). The clusters of bacterial and fungal taxa among different time points were more separate in the rhizosphere of LJ43 than in ZC108 at different time points (Fig. 2a). Moreover, fungi showed more separate clustering among different time points than bacteria in the rhizosphere of LJ43 and ZC108 (Fig. 2b). Bacterial classes in the rhizosphere varied in the relative abundance between the tea cultivars, including Actinobacteria, Acidobacteriae, Thermolephilia, Bacilli, Ktedonobacteria, and Verrucomicrobiae. The sampling time affected the relative abundance of Actinobacteria, Alphaproteobacteria, Acidobacteriae, Ktedonobacteria, Acidimicrobiia, Actinobacteriota, and Verrucomicrobiae classes (Fig. 2c). In fungi, the classes of Tremellomycetes, Sordariomycetes, Rozellomycota, and Incertae sedis showed clearly different abundances between tea cultivars and sampling times, and their interaction also affected the fungal composition (Fig. 2d). LJ43 showed a slightly higher abundance of bacterial classes, including Actinobacteria, and of some fungal classes, including Rozellomucota and Eurotiomycetes, in the rhizosphere (Fig. S2). The rhizosphere bacterial classes Bacilli and Acidobacteriae, as well as fungal classes Tremellomycetes and Sordariomycetes, exhibited higher abundances in ZC108 than in LJ43 (Fig. S3). ZC108 had higher abundances of Actinobacteria, Gammaproteobacteria, Thermolephilia, Acidimicrobiia, and TK10 classes (bacteria), and of Rozellomucota and Incertae sedis classes (fungi) at midday than at midnight (Figs. S3, S4, S5). In contrast, the bacterial classes Acidobacteriae and fungal classes Tremellomycetes were more abundant at midnight relative to midday in the rhizosphere of ZC108 (Figs. S3, S4, S5). There was no significant difference in bacterial classes between midday and midnight in the rhizosphere of LJ43 (Figs. S3, S4). LJ43 showed increased levels of the fungal class Sordariomycetes at midnight, while the fungal classes Dothideomycetes and Incertae sedis were more frequent at midday than at midnight (Fig. S6).

### Circadian rhythms of microbial communities in the rhizosphere

**Figure 3:**
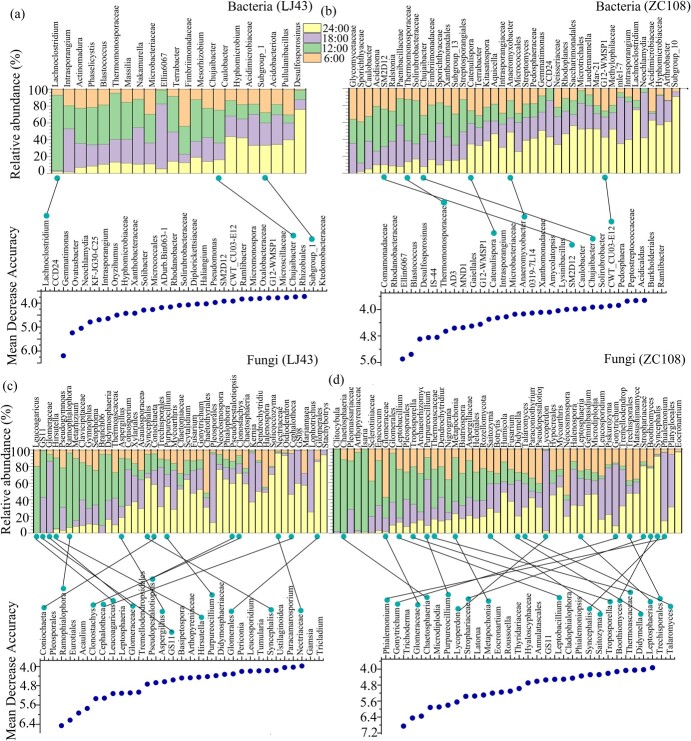
Bacteria (a, b) and fungi (c, d) in the rhizosphere with identified circadian rhythms (up) and the top 29 taxa in ‘Longjing43’ (LJ43) (a, c) and ‘Zhongcha108’ (ZC108) (b, d). The upper heat map shows the genera, which were selected based on the relative abundance >0.1% and the abundance folds >2 for differences between midday and midnight.

The relative abundances of rhizosphere fungal genera were more easily affected by the diurnal cycling than those of bacterial genera (>0.1%): 71% and 70% of the fungal communities (45 and 50 genera) showed midday–midnight variations in the rhizosphere of LJ43 and ZC108, respectively, while only 35% and 61% (22 and 42 genera) of bacterial communities showed a midday–midnight schedule in the rhizosphere of LJ43 and ZC108, respectively (Fig. 3a, b). Similarly, fungi had more genera with circadian rhythms than bacteria: 22% and 25% (14 and 18 genera) of fungal communities, and 5% and 9% (3 and 6 genera) of bacterial communities in the rhizosphere of LJ43 and ZC108, respectively (Fig. 3c, d). Of the genera with identified circadian rhythms, only the bacterial genus Chujaibacter was shared by LJ43 and ZC108, and the fungal genera of Glomeraceae, and Purpureocillium and Syncephalis were shared by LJ43 and ZC108.

### Nutrient changes in the rhizosphere related to N and P

**Figure 4:**
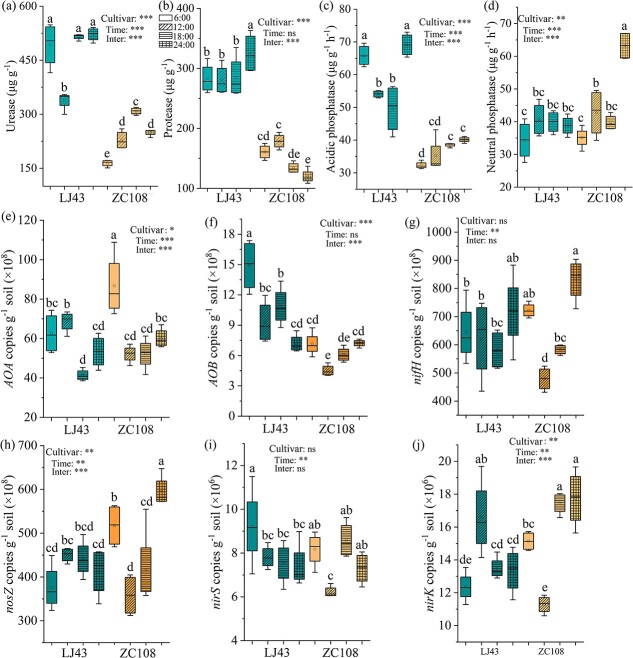
Urease (a), protease (b), acidic phosphatase (c), and neutral phosphatase (d) activities, and the absolute abundance of genes relative to nitrogen nitrification (*AOA* and *AOB*) (e, f), fixation (*nifH*) (g), and denitrification (*nirS*, *nirK*, and *nosZ*) (h, i, j) in the rhizosphere of ‘Longjing43’ (LJ43) and ‘Zhongcha108’ (ZC108). Different lower-case letters above bars indicate significant differences among treatments at *P* < 0.05 based on ANOVA followed by Duncan’s tests. cultivar, tea cultivar main effect; Time, time main effect; Inter, interactive effect of tea cultivar and time. ^*^0.01 < *P* ≤ 0.05; ^**^0.001 < *P* ≤ 0.01; and ^***^P ≤ 0.001. ns, no difference.

Differences between the tea cultivars in urease, protease, acidic phosphatase, and neutral phosphatase activities were found to be dependent on rhythmicity; LJ43 had higher activity levels than that of ZC108, except for neutral phosphatase activity (Fig. 4a–d). The activities of urease, protease, and acidic phosphatase were higher at midnight than that at midday; however, no difference in neutral phosphatase activity was observed between midnight and midday in the rhizosphere of LJ43. ZC108 had higher neutral phosphatase activity but lower protease activity in the rhizosphere at midnight than that at midday (Fig. 4b, d); however, no difference in the activities of urease and neutral phosphatase was observed between these two time points (Fig. 4a, c). The two tea cultivars showed differences in the expression of some genes related to N cycling that were dependent on diurnal cycling (Fig. 4e–j). The genes *AOA* and *nirK* showed greater expression at midday than at midnight in LJ43, while the other genes showed similar levels at these two time points in LJ43 (Fig. 4e, j). In ZC108, the N cycling was much stronger at midnight than that at midday, as shown by the higher expression levels of N cycling-related genes, except for *AOA,* which had the highest value in the morning. Furthermore, the diurnal cycling in the expression of N cycling-related genes was more dynamic in the rhizosphere of ZC108 than that in LJ43, and the genes *nifH*, *nosZ*, and *nirK* had higher expression levels in the rhizosphere of ZC108 than that in the rhizosphere of LJ43.

### Correlations between bacterial and fungal communities and soil properties

**Figure 5:**
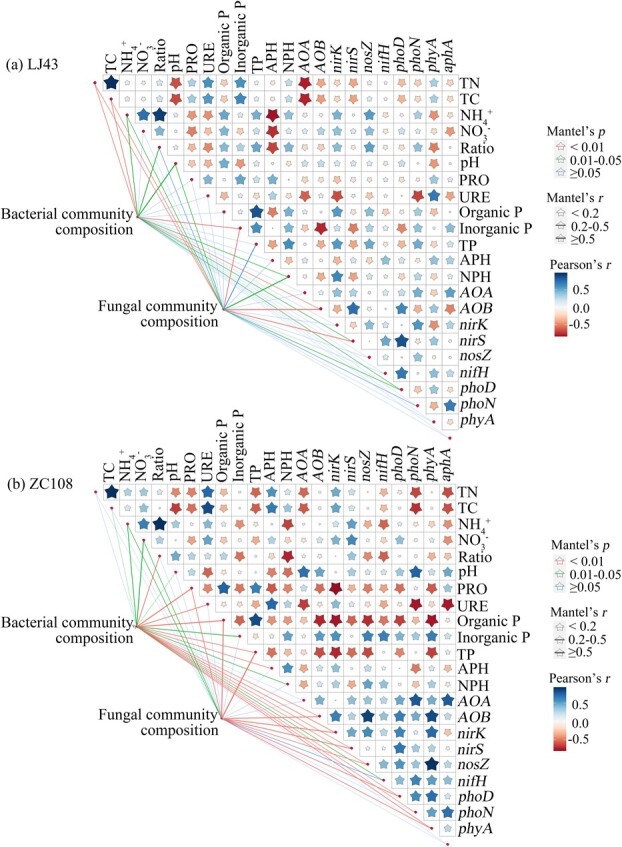
Correlations of soil physiochemical properties and rhizosphere bacterial and fungal community compositions in ‘Longjing43’ (LJ43) (a) and ‘Zhongcha108’ (ZC108) (b). The dissimilarity matrix of bacterial and fungal communities was calculated with the Bray–Curties distance, and the matrix of soil physiochemical properties (Table S1) was calculated with the Euclidean distance. The line width represents the Mantel’s r value and the line color represents the corresponding *P-*value. TN, total nitrogen; TC, total C; ratio, the ratio of ammonium (NH_4_^+^) to nitrate (NO_3_^−^); PRO, Protease; URE, urease; organic P, organic phosphorus; inorganic P, inorganic phosphorus; TP, total phosphorus; APH, acidic phosphatase; NPH, neutral phosphatase; *AOA* and *AOB*, genes related to nitrification; *nirK*, *nirS*, and *nosZ*, genes related to denitrification; *nifH*, gene related to nitrogen fixation; *phoD* and *phyA*, genes related to alkaline phosphatase activity; *aphA* and *phoN*, genes related to acidic phosphatase activity. . The gene primers were given in Table S2.

RDA was performed to explore how the soil properties affect the composition of fungal and bacterial communities (Fig. 5a, b). The correlations were clearer in the rhizosphere of ZC108 than in the rhizosphere of LJ43. Briefly, in LJ43, the composition of bacterial communities was correlated with soil total C, N, inorganic P, and *AOB*, while the composition of fungal communities was associated with total C, N, inorganic P, *AOB*, and *nirS* (Fig. 5a, b). In contrast, the composition of bacterial communities exerted great effects on diurnal variations in organic P and N mineralization via activating protease activity, urease activity, organic P, and total P, and also the genes related to N nitrification and denitrification, including *AOB*, *nirK*, *nirS*, *nosZ*, *phoD*, *phoN*, and *phyA* in the rhizosphere of ZC108 (Fig. 5a). The composition of fungal communities was connected with protease activity, organic P, total P, *AOB*, *nirK*, *nirS*, *nosZ*, *phoD*, *phoN*, and *phyA* in the rhizosphere of ZC108 (Fig. 5b). RDA results showed the diurnal changes in bacterial and fungal community compositions showed greatest effects on soil organic P and inorganic N cycling in the rhizosphere of ZC108.

## Discussion

### Alteration in bacterial and fungal rhythmicity in the rhizosphere of tea cultivars

**Figure 6:**
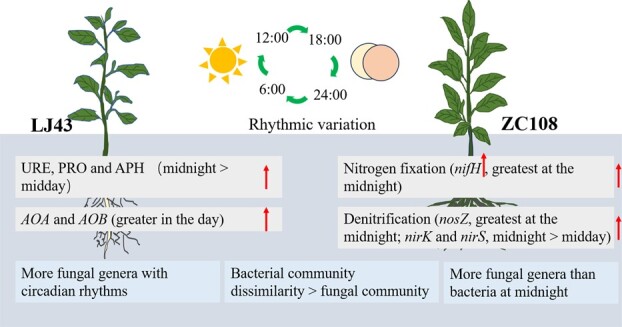
Schematic diagram of the role of the circadian clock of plants affecting the composition and ecological function of bacterial and fungal communities in the rhizosphere of ‘Longjing43’ (LJ43) and ‘Zhongcha108’ (ZC108). Arrows indicate a stronger rhythmic variation in the rhizosphere of tea plant cultivars. *AOA* and *AOB*, genes related to nitrification; *nirK*, *nirS*, and *nosZ*, genes related to denitrification; *nifH*, gene related to nitrogen fixation; PRO, protease; URE, urease; APH, acidic phosphatase activity.

The composition of bacterial and fungal communities in the rhizosphere of tea plants showed diurnal cycles, as indicated by the rhythmic changes in their relative abundance (Figs. 1–3; Fig. 6). Here, the relative abundances of most bacterial genera at midday and midnight are dependent on the tea cultivar and show a higher abundance at midday for LJ43 and at midnight for ZC108. Plants’ circadian rhythm regulates the microbial community composition, and the misfunction of the circadian clock in host plants leads to changes in the assembly of the rhizosphere microbiome [2, 5]. The bacteria and fungi responded differently to the diel rhythm of day–night cycling, as the composition of fungal communities was less affected by rhythm (a lower ecological niche overlap) when compared to that by the bacterial communities in the rhizosphere of LJ43 and ZC108 (Fig. 2). This finding is consistent with that of the earlier reports showing that fungal communities are more resistant to environmental disturbances than bacterial communities [40, 41], which is likely due to their specific physiological features, such as aerial hyphae and spore production [42]. Fungi often prefer root litter-deprived C sources and specific physiological features (such as low nutrient demands), which intensify the utilization of root-derived C and their tolerance to harsh environments relative to bacterial communities [43]. It was reported that soil fungal networks are more stable than bacterial networks under drought and permafrost degradation conditions [40, 41]. A more stable network in the fungal community implies a lower ecological niche overlap, and, therefore, fungal taxa respond differently to changing environmental conditions [41, 44]. In fact, we found that fungal genera with circadian rhythms were more frequent than that of bacterial taxa, and the fungal community was more stable with a lower network degree than that of the bacterial community, irrespective of the tea cultivar (Fig. 3, Fig. S7). Some dominant fungal classes also showed significant differences in abundance under different light/dark schedules (Fig. 3). Fungal circadian rhythms are largely controlled by circadian rhythms, such as spore development and release [45]. More frequent fungal taxa with a diel rhythm in the rhizosphere relative to bacterial taxa may be a result of their ability to maintain dominance and community network stability under the day–night schedule (Fig. 3, Fig. S7), as shown by earlier results [40].

Fungi can colonize 80% of plant species and receive C from host plants to transport nutrients absorbed from the soil [46, 47]. Plant rhythmicity in available C metabolism, transport, and storage exerts a greater effect on fungal growth and dieback than that on bacterial taxa [48–50]. Plants accumulate non-structural carbohydrates during the daytime and remobilize them at night to support plant growth and respiration [51, 52]. Most fungal species respond to light, unlike bacteria [7]. Fungal taxol production was enhanced [53], which may explain the high abundance of most fungal genera at midnight (Fig. 3). The significantly higher soil moisture at midnight may also promote mycelial growth [54]. Therefore, fungal genera may exhibit more pronounced circadian rhythms in the rhizosphere than that of bacterial genus. Plants may exhibit a diurnal rhythmicity in nutrient uptake and show that the highest peak occurred with highest transpiration, although another peak at night was also observed [55]. Diurnal fungal genera may adjust the rhythmicity of plant nutrient acquisition and availability [50]. The close correlation between fungal community components and soil nutrient traits may partly explain the possible role of fungal taxa in plant nutrient availability.

Internal circadian rhythms are subject to extensive variation among different plants and genotypes [5, 23], which also caused dynamic changes in microbial composition and abundance in the rhizosphere throughout the day–night cycle [2]. The rhizosphere community composition and abundance of bacteria and fungi exhibited cultivar-dependent rhythmic variations under light/dark schedules (Figs. 1–3). LJ43 showed more extensive variation in bacterial and fungal communities in the rhizosphere at the four time points compared to that of ZC108 (Fig. 2). ZC108 and LJ43 showed different leaf shapes, bud colors, and plant architecture, and LJ43 showed higher transpiration rates, lower water use efficiency and photosynthetic ability than ZC108 [25, 37]. The diversity of plants’ functional traits due to adaptation to different environmental factors is expected to lead to novel host traits that produce new niches for microbial colonization [56]. The rhythmic responses of plants can cause diurnal changes in soil oxygen, nutrients, and pH [3, 20]. LJ43 and ZC108 showed cultivar-specific responses to light intensity, light quality, nutrient acquisition, and utilization, and large differential genes were induced in response to abiotic and biotic stressors [24, 57, 58]. Diverse bacterial taxa have different nutrient preferences depending on the availability of resources, which can lead to differences in growth rates under different nutrient conditions [59]. The different responses of tea cultivars to circadian rhythms provide divergent microhabitats, leading to varying responses among bacterial taxa to day–night rhythmicity in their abundance. The rhythmicity of leaf photosynthesis, C transport, and metabolism leads to diurnal changes in root exudate profiles that could induce circadian alteration in the microbiome. The circadian rhythm of root exudate profiles is determined by plant species [60], which may explain the variation of the diel rhythm in the microbial communities in their rhizosphere. The altered root exudates also induce the movement of some bacterial taxa through water-filled spaces in the soil at particular times of the day [60]. Rhythmic plant defense against microbial pathogens induces the production of many signaling molecules [61]. Differentially expressed genes in the leaves of LJ43 and ZC108 were involved in secondary metabolism, hormone biosynthesis, and signaling [24]. These differences in the morphological, physiological, and molecular traits between LJ43 and ZC108 may explain the different patterns of microbial rhythmicity.

Most fungal genera in the rhizosphere were more abundant in darkness, regardless of the tea cultivar (Fig. 3). ZC108 possessed more genera with circadian rhythms in the rhizosphere than that of LJ43, suggesting that bacterial and fungal community variation under light–dark schedules was not consistent with the number of taxa with circadian rhythms. In this study, we identified microbiomes with circadian rhythms based on an analysis using the random forest model. The abundances of bacterial and fungal genera were analyzed using the midnight and midday data (Fig. 3). The random forest model showed only the first 20 fungal or bacterial taxa; some rare microbial taxa were excluded. Circadian rhythms in plants largely influence the structure and function of rare taxa [62, 63]. These results may explain the inconsistency in the extent of variation in community composition and the number of genera with circadian rhythms (Figs. 2, 3).

### Alterations in the rhythmicity of microbial-mediated rhizosphere nutrient processes in tea cultivars

The rhythmicity of the internal clock shows extensive natural variations within and between species [64], and enables organisms to adapt to different environments by altering their metabolic and ecological function [3, 7]. Rhizosphere nutrient cycling was controlled by light/dark schedules, depending on the tea cultivar (Figs. 4, 6). The diurnal rhythmicity of organic N and P mineralization was more significant in the rhizosphere of LJ43, as evidenced by the increased enzymatic activity related to organic N and P mineralization (urease and acidic phosphatase) (Figs. 4, 6). The rhythmicity of organic matter composition reflects the possibility of organic matter mineralization and biosynthesis [2]. Soil protease and urease catalyze organic matter mineralization and release available N [65, 66], whereas phosphatase hydrolyzes organic P to release inorganic P [67]. Enzymes related to organic N and P mineralization were higher in the rhizosphere of LJ43 at midnight, whereas the rhizosphere protease activity of ZC108 was greater during the daytime (Fig. 4). The increased protease and acidic phosphatase activities in the rhizosphere of LJ43 may facilitate N and P mineralization in the dark and provide sufficient available N and P to the plants during the day.

The organic P and N mineralization is principally catalyzed by extracellular enzymes produced by soil microorganisms [68]. The upregulation of predicted genes related to organic matter mineralization in the rhizosphere of LJ43 also supports these findings (Fig. S8). The abundance of bacterial and fungal genera with identified circadian rhythms increased at night in LJ43, especially some saprophytic microbiomes, implying the potential for microbial-mediated mineralization of organic matter in the dark. Genes related to carbohydrate metabolism are more abundant in dark samples [2], which underlies the possible role of microbiome in organic nutrient mineralization in the dark. Bacterial and fungal taxa in the rhizosphere also showed diel rhythmicity, particularly in ZC108 (Figs. 1–3). Furthermore, the composition of the bacterial and fungal communities exhibited a close correlation with protease and phosphatase changes in the rhizosphere of ZC108, even though greater diel variation was present in the enzymatic activities of LJ43 (Fig. 5), indicating the potential for microbial-mediated organic N and P mineralization. Acidobacteria members are reported to regulate nutrient cycling and organic matter decomposition and tolerate a wide range of soil pH values [69, 70]. Here, we found that Acidobacteria were significantly correlated with soil N and P cycling in the rhizosphere of ZC108, suggesting their potential to regulate soil nutrient cycling in acidic tea plantation soils. Additionally, a strong correlation between the Gammaproteobacteria and Acidobacteria phyla and soil nutrients was found in the soils of ZC108. Gammaproteobacteria and Acidobacteria phyla are also involved in nutrient cycling, plant growth, and crop production [71, 72], which may regulate the nutrients available in the soils of tea plantations.

Nevertheless, methodological limitations may have affected the interpretation of nutrient cycling rhythmicity. Some microbial taxa showed diurnal responses to light and time points, and the extraction time of soil samples and light intensity in the laboratory may have affected the rhythmicity of enzyme activities. Additionally, the rhizosphere is regarded as a rhythmic environment; diel oscillations in soil pH, nutrients, oxygen, and water potential affect the microbial migration into the rhizosphere, and their abundance and functions [2, 20]. The measurement of enzyme activities *in vitro* cannot completely reflect the rhythmic rhizosphere environment, which may limit the interpretation of rhythmicity of nutrient cycling. Therefore, method optimization is required to estimate the rhythmicity in nutrient cycling in future studies. Noticeably, LJ43 and ZC108 are widely cultivated in Zhejiang province. The selection of sampling times and locations can represent the broader ecological conditions of tea plantations in this region. We did not exclude the possible difference in response of the rhizospheric microbiome to dial changes in other regions with different climatic conditions or management practices. This needs to be explored in future studies.

Denitrification and nitrification involve the reduction of nitrate and nitrite to N_2_O and N_2_ catalyzed by enzymes encoded by *nirS*, *nirK*, and *nosZ* genes, and ammonia oxidation catalyzed by ammonia-oxidizing archaea (AOA) and ammonia-oxidizing bacteria (AOB), respectively [73]. Here, denitrification and nitrification also showed diel rhythmicity along with day–night schedules in the rhizosphere of ZC108 and LJ43, and that nitrification in the morning and denitrification at midnight reached the greatest values, as evidenced by *AOA* and *AOB* gene expression (Fig. 5). Functional microbial genes related to N cycling are controlled by the light and dark conditions [31]. Nitrifying bacteria, including AOA and AOB, are often sensitive to light, and strong light inhibits their growth [74]. Furthermore, microbial metabolism depends on electron transfer with the supply of oxygen, and low oxygen concentrations at night would decrease microbial C catabolic and metabolic processes [75]. These earlier results may explain the intensive expression of *AOA* and *AOB* genes and the high nitrification potential in the morning.

Diel rhythms also affect N fixation and denitrification processes mediated by the rhizosphere microbiome. The most commonly used denitrification-related genes include *nirS/K* for nitrite reduction and *nosZ* for N_2_O reduction [76]. Rhizosphere denitrification may contribute to microbial circadian clocks, as the genes related to denitrification, *nirS*, *nirK*, and *nosZ*, varied along the light–dark schedule, and this rhythmicity was greater in ZC108 than that of LJ43 (Figs. 4, 6). Genes related to denitrification (*nosZ*, *nirK*, and *nirS*) peaked at midnight in the ZC108 rhizosphere, suggesting that microbially regulated denitrification may proceed in the dark. Consistently, some microbiomes exhibit daily rhythms in nitrous oxide emission in oilseed rape under field conditions, peaking in the afternoon [32]. Metabolic activity for N removal was found to be greater at night than during the daytime [31]. The *nosZ* genes are dominant in the process of denitrification in acidic tea plantation soils, as evidenced by an order of magnitude higher number of gene copies of *nosZ* relative to *nirK* and *nirS* in the rhizosphere of ZC108, suggesting intensive *nosZ*-mediated N_2_O emissions. Contrastingly, microbially regulated denitrification showed fewer diurnal changes, as shown by the expression patterns of *nosZ*, *nirK*, and *nirS* in the rhizosphere of LJ43. Biodiversity and multifunctionality in a microbial community may cause redundancy in microbial function and different responses to environmental disturbances. LJ43 and ZC108 have different bacterial and fungal abundances and responses to the soil environment [38, 39], which may account for the dissimilarity in denitrification in the rhizosphere. The rhizosphere microbial community composition showed a strong correlation with the daily changes in inorganic N cycling in ZC108, which may have led to greater susceptibility to circadian rhythms than in the tea cultivar LJ43 (Fig. 5). Dynamic changes in microbial taxa-driven inorganic N cycling may pose a great risk for N loss in ZC108 plantations. Examination of the mechanism of rhizosphere plant–microbial interactions may help develop new strategies to enhance the sustainable development of tea gardens.

N fixation is regulated by the conserved subunit of the dinitrogenase iron protein, which is encoded by the *nifH* gene, a genetic marker of N-fixing bacteria [77], and by a circadian rhythm [6, 32]. Specifically, the rhizosphere microbiome-regulated N fixation of ZC108 reached its highest value at midnight, and its ability was greater than that of LJ43, as evidenced by the high copy numbers of the *nifH* gene in ZC108. The high relative abundance of N-fixed bacterial taxa in the rhizosphere of ZC108 also supports the potential for N_2_ fixation at midnight. Nitrogenase is synthesized daily and exhibits peak abundance during dark periods [6]. Additionally, the transformation of inorganic P pools was affected by the day–night schedules, as evidenced by the rhythmicity in Ca-P (Table S3). Soil P is a major limiting factor for plant growth and development due to the low slow diffusion and high fixation in soils. Most of the soil P is locked in the moderately occluded minerals and recalcitrant P minerals [78]. Therefore, the enhancement of insoluble P transformation to available P and the maintaining of a level availably P supply can maximize the root acquisitive efficiency [79–81]. Carboxylates released by the roots are critical in mobilizing precipitated phosphates in acidic soils [82]. The diel rhythmicity of the quality and quantality of root exudates may explain the diurnal variation of Ca-P contents. The induced small-molecule carboxylic acids and decrease soil pH enhanced the inorganic mobilization [72]. Root exudate profile and rhizosphere pH also vary depending on the plant species, genotypes, and rhizosphere environment. Genetic variation and environmental conditions may change the rhythmicity of C biosynthesis, transport, and organic acid secretion by roots [83, 84]. Additionally, the rhythmicity of root exudate profiles influences diel changes in rhizosphere pH and microbial community structure and abundance [2, 20], which may change P availability and needs to be further explored.

Knowledge of microbial rhythmicity can help to develop novel chronobiology-based strategies for crop management. Plants exhibit a rhythmic defense responses against pathogens, and clock-based strategies influence plant resistance and growth. The rhythmic variation in the rhizosphere microbiome also increases the success of soil-based agricultural interventions [26], such as microbial inoculant establishment, via changes in application time, and the rhythmic demand for nutrients.

## Conclusion

Our results concluded that the microbial rhythmicity changed the temporal pattern of rhizosphere biochemical processes and affected nutrient availability. Fungal genera with circadian rhythms were more abundant than bacterial communities, especially in the rhizosphere of LJ43. Most fungal genera showed higher relative abundances in the darkness. Organic N and P mineralization were more intensively affected by diel changes in the rhizosphere of LJ43. In contrast, microbiome-mediated N fixation and denitrification potential were the greatest at midnight in the rhizosphere of ZC108. The knowledge of circadian on soil microbial composition and diversity could provide a novel agricultural chronotherapy for soil management and tea production practices. Further work is needed to uncover how microbial functions are coordinated with the plant transcriptome in improving tea yield or quality. It is also necessary to identify the rhythmicity of core microbial taxa and genes and explore their roles in regulating soil nutrient availability and plant nutrient acquisition in the future.

## Materials and methods

### Field experiment design and sampling

The experiment was performed in the tea gardens of the Shengzhou integrated experimental station of the Zhejiang Province, China (120°48′E, 29°75′N). This region belongs to the subtropical monsoon climate, with an annual mean temperature of 16.0°C–17.5°C, ~900–1500 mm of annual precipitation and an annual sunshine time of 1100–2200 h. LJ43 and ZC108 plants have been cultivated on both sampling sites since 2002 under similar management practices. The soil properties of the tea plantation are given in Table S1. We randomly established four sampling plots with 6 × 6 m per tea cultivation. Sampling was performed at 6:00, 12:00, 18:00, and 24:00 during three consecutive days in the middle of March 2022. Plant responses to diurnal changes in light lead to physiological adaptation, such as photosynthesis and respiration, as well as rhizospheric microenvironment [2, 12, 18]. This time scheme represents the key time point of plant photosynthesis and potential, as well as the diurnal changes in light. Moreover, the diurnal rhythms of environments reflected the change in the light–dark cycle of a day. The high-quality tea plucking of tea bud is between late February and mid-March. The seasonal variations may also influence the diurnal rhythms, but we mainly help to develop novel chronobiology-based strategies for tea production practices. On each plot, we randomly selected six tea plants as one replicate within a plot. Therefore, we produced a total of 96 samples (2 tea cultivars × 12 sampling times × 4 replicates). Rhizosphere soil samples were collected from each tree after separating roots from the soils. Soil samples used to physiochemical traits were transported into the laboratory with ice and samples used for microbial sequencing were transported into the laboratory with dry ice. One part of the rhizosphere soil sample was air-dried and sieved (2 mm) for the measurement of soil physiochemical traits. One part of the sample transported with dry ice was stored at −80°C until analysis.

The soil pH was measured using glass electrodes with a PHBJ-260 pH meter (Shanghai Leici, China) [85]. Soil total C and N were measured with a C and N analyzer (Multi C/N 3100; Jena Analytics, Jena, Germany). The total P content in soil was measured with the molybdenum blue colorimetry [86]. The soil samples were extracted with 1 M KCl and the contents of soil NO_3_^−^ and NH_4_^+^ were measured with a flow analytical system (SEAL Analytical Auto Analyzer 3) [87]. The levels of microbial C and N were measured with chloroform fumigation. Briefly, the fresh soil samples were fumigated with chloroform and extracted with 0.5 M K_2_SO_4_ (1:5 ratio of soil to solution). The non-fumigated soil samples were used as the control. The C and N contents of the solution were measured with a C and N analyzer (Multi C/N 3100; Jena Analytics, Jena, Germany). The P content of the solution was measured with molybdenum blue colorimetry. The microbial C, N, and P contents were calculated as the differences of C, N, and P contents in the solution between fumigated and non-fumigated samples.

### Soil enzyme activities

The soil protease activity was measured as follows: a total of 2 g of soil samples was mixed with the extraction solutions containing 2.5 ml of 0.2 M Tris buffer (pH 8.0) and 2.5 ml of Na-caseinate solution (2%), and the solution was subsequently incubated at 30°C for 2 h. The mixture was added to 5 ml of trichloroacetic acid (10%) and centrifuged for 10 min. The supernatant was mixed with 0.75 ml of Na_2_CO_3_ and 0.25 ml of the Folin–Ciocalteu reagent and measured colorimetrically at 680 nm. For soil urease, 2-g soil samples were extracted by the extraction solutions containing 1 ml methylbenzene, 10 ml urea (10%), and 20 ml citric acid buffer (pH 8.0). The mixed solution was shocked and incubated at 37°C for 24 h. A total of 2 ml of the filtrate was mixed with 4 ml phenol and 3 ml sodium hypochlorite and reacted 20 min in the dark. The reaction solution was subsequently measured colorimetrically at 578 nm [88]. For phosphatase activity [89], 2 g of soil samples were added into 0.5 ml methylbenzene, 4 ml of 150 mM p-nitrophenyl phosphate solution, and then buffer solution (200 mM CH_3_COONa (pH 5.2) for the acid phosphatase activity measurement; 100 mM NaHCO_3_ (pH 7.0) for the neutral phosphatase activity measurement). After incubating for 24 h at 30°C, the reaction was stopped by 0.5 M NaOH and determined at 400 nm.

### Soil P fraction

Different soil P fractions were determined as follows [90]: (1) resin-P: soil samples were extracted with 1 g resin to obtain water-soluble P; (2) NaHCO_3_-P (Ca_2_-P): the above pellets were extracted by 30 ml of 0.5 mol l^−1^ NaHCO_3_ (pH 8.5) and centrifuged at 4500 g for 10 min at 4°C. A total of 10 ml of clear supernatant was absorbed and reacted with 6 ml of 0.9 mol l^−1^ H_2_SO_4_. The NaHCO_3_-extracted inorganic P_i_ was measured using the molybdenum antimony colorimetry. The other part of supernatant (5 ml) was mixed with 0.5 g of (NH_4_)_2_S_2_O_8_ and 10 ml of 0.9 mol l^−1^ H_2_SO_4_ and thus autoclaved for sterilization for 1 h. The reactive solution was used to measure the NaHCO_3_-extracted total P_t_ using the molybdenum antimony colorimetry. The NaHCO_3_-extracted organic P_o_ was calculated as the difference between total P in the form of NaHCO_3_ and inorganic P in the form of NaHCO_3_. (3) NaOH-P (Al, Fe-binding P): the above pellets were extracted by 30 ml of 0.1 mol l^−1^ NaOH and centrifuged at 25 000 g for 10 min at 4°C. The clear supernatants (10 ml) were mixed with of 1.6 ml 0.9 mol l^−1^ H_2_SO_4_ for measurement of NaON-extracted total P NaOH-P_t_. The other supernatant was autoclaved for 1.5 h and used to measure the NaON-extracted inorganic P NaOH-P_i_. The NaOH-extracted organic P_o_ was calculated as the difference between total P in the form of NaOH and inorganic P in the form of NaOH. (4) HCl-P (Ca_8_-P and Ca_10_-P): the pellets produced above were extracted by 10 ml of HCl. Each produced solution was measured by molybdenum antimony colorimetry. The residual P consisted of refractory organic matter and minerals, and it was calculated as the difference between soil total P and the first four fractions of P.

### 16S rRNA gene sequencing and bioinformatic analysis

About 50 mg of rhizosphere soil samples were used to extract bacterial and fungal DNA with a Power Soil DNA Isolation Kit (MO BIO Laboratories, Inc. Carlsbad, CA, USA) according to the manufacturer’s instructions. The 16S rRNA gene of the V3-V4 region of bacteria was amplified using the primers 338F (5’-ACTCCTACGGGAGGCAGCAG-3′) and 806R (5’-GGACTACHVGGGTWTCTAAT-3′). The ITS2 region (fITS7/ITS4) of fungi was amplified using the primers ITS3F (5’-GCATCGATGAAGAACGCAGC-3′) and ITS4R (5’-TCCTCCGCTTATTGATATGC-3′). The polymerase chain reaction (PCR) was performed in a 50-μl reactive system containing 3 μl template DNA, 25 μl 2× Premix Taq, 1 μl of 10 μM each primer and 21 μl nuclease-free water with the following condition: 95°C for 5 min; 30 cycles of 95°C for 30 s, and 56°C for 30 s; an extension phase of 72°C for 30 s; and 55°C to 95°C for melting curve analyses. The PCR reaction was performed with a PCR instrument (Bio-Rad Laboratory, CA, USA). The library was sequenced using an Illumina MiSeq instrument (Illumina, San Diego, California, USA), and paired-end reads of 250 bp were generated. The quality of raw data was controlled with the Fastp (version 0.14.1) sliding window. The paired-end clean reads were obtained after removing the primers by using cutadapt software and merging with search-fastq mergepairs. The ASVs were screened based on 97% nucleotide identity with UPARSE [91]. An average of 184 350 bacterial and 11 064 fungal ASVs were produced for subsequent analyses using UNITE (V.8.0) and SILVA (v.138) as reference databases, respectively.

### Real-time q-PCR analysis of N-related genes

The extracted DNA from soil samples was quantified by real-time q-PCR according to the manufacturer’s instructions (Vazyme Biotech Co., Ltd, Nanjing, China). The used primers are given in Table S2. The reaction mixtures contained 5 μM primer (0.8 μl of forward and reverse primer, respectively), 10 μl of 2× SYBR Color qPCR Master Mix, 0.4 μl ROX Reference Dye, 2 μl of template DNA, and 6 μl of ddH_2_O. The following conditions were used: initial denaturation at 95°C for 5 min, at 95°C for 3 min for 40 cycles, melting at 95°C for 5 s, annealing at 58°C for 30 s and extension at 72°C for 1 min.

### Statistical analysis

After checking the normality of the data, we performed the statistical analysis with SPSS software (version 22) followed by Duncan’s test (version 22.0) (*P* < 0.05). The α-diversity indices of bacterial and fungal communities were calculated with R (V3.5.3) and the relative figure was plotted by the ‘*ggplot2*’ package. The β-diversity indices of communities were calculated with the non-metric multi-dimensional scaling (NMDS) according to the Bray–Curtis dissimilarities distance matrix of the Phyloseq package [92]. Classes with a relative abundance of >1% were defined as the dominant class and classes with an abundance <1% were combined as ‘others’. Genera with statistically significant differences in normalized abundance between day and night, which also displayed the same change trends between day and night, were regarded as taxa with circadian rhythms. Genera with 2-fold differences in the relative abundance between midday (12:00) and midnight (24:00) were regarded as taxa with circadian rhythms. The differential bacterial and fungal taxa were identified based on the thresholds of >2-fold changes (*P* < 0.05). A random forest analysis was conducted to identify the top 29 important genera with responses to day–night cycling using the RANDOMFOREST package in R package [4]. The correlations of soil physiochemical properties and the microbial community composition in the rhizosphere were assessed with Pearson’s correlations and Mantel test and visualized by the ‘*ggcor*’ package in R [93]. Phylogenetic trees were constructed with the maximum likelihood and neighbor-joining methods and visualized in iTOL software.

## Supplementary Material

Web_Material_uhae267

## Data Availability

Raw DNA sequence files and associated metadata were deposited in the NCBI data bank with the accession number PRJNA1010133. All records of statistical analyses are included in Supplementary Materials2. They were created with the SPSS software (version 22).
