# Supplementary material for: Circadian rhythms of microbial communities and their role in regulating nitrogen and phosphorus cycling in the rhizosphere of tea plants
Source: Hortic Res. 2024 Oct 9;12(1):uhae267. doi: 10.1093/hr/uhae267 (PMC11718400; doi:10.1093/hr/uhae267)
Supplement: Web_Material_uhae267 [file web_material_uhae267.zip › HR-2024-375.R2_Supplementary Materials 1.docx]

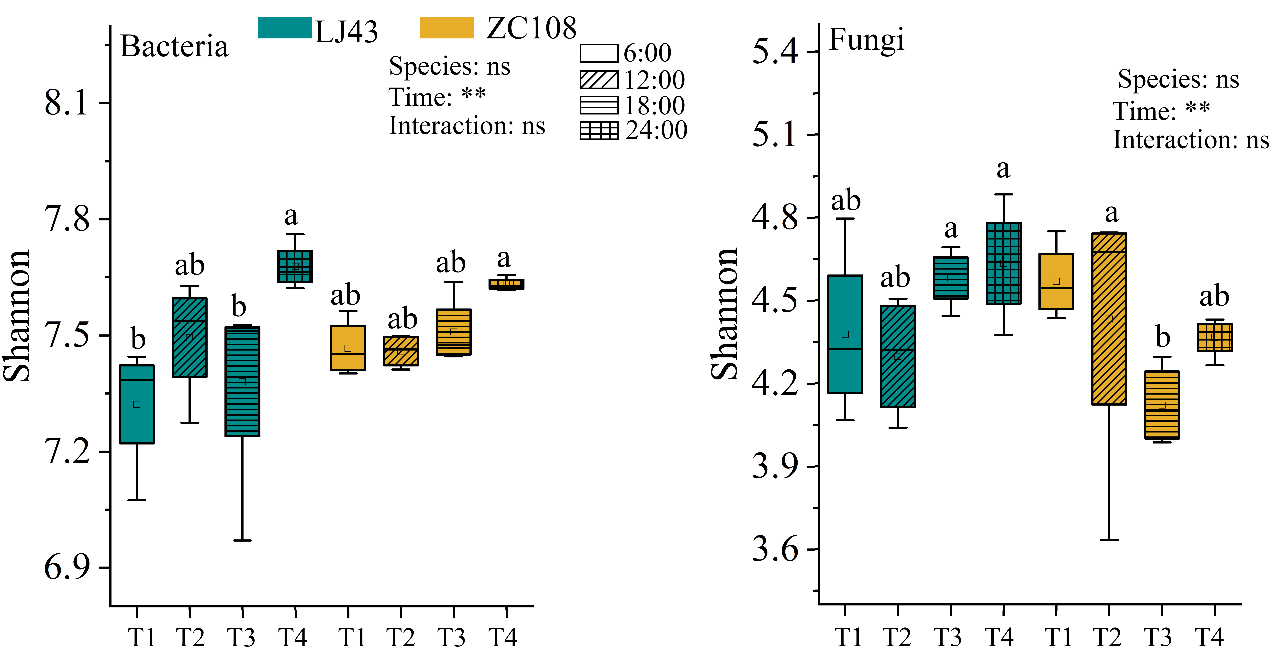


**Figure S1** The α diversity indexes (Shannon) of bacterial and fungal communities in the rhizosphere of two tea species ‘Zhongcha108’ (ZC108) and ‘Longjing43’ (LJ43) at different times of the day. Different lower-case letters above bars indicate significant differences among treatments at *P* < 0.05 based on ANOVA followed by Duncan’s tests. T1, 6:00; T2, 12:00; T3, 18:00; T4, 24:00. Species, tea species main effect; time, time main effect; Interaction, interactive effect of tea species and time. * 0.01< *P* ≤ 0.05; ** 0.001< *P* ≤ 0.01; and ****P* ≤ 0.001. ns, no difference.


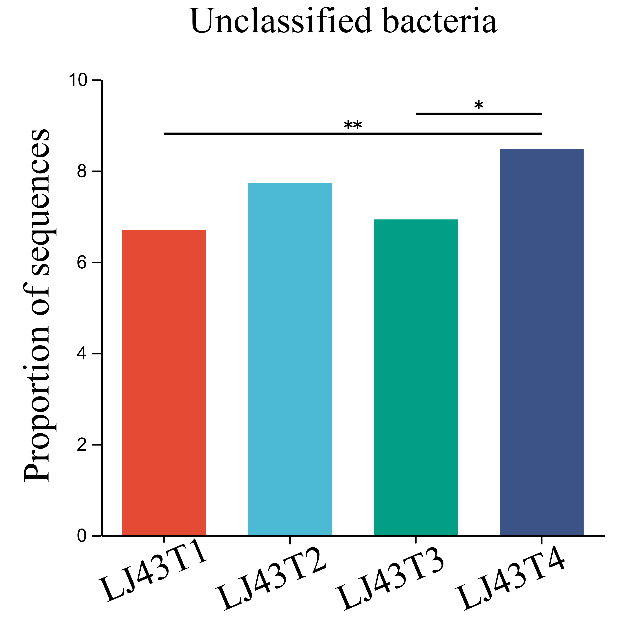


**Figure S2** The dominant bacterial classes in the rhizosphere of tea plants ‘Longjin43’ (LJ43) at different times of the day. Different lower-case letters above bars indicate significant differences among treatments at *P* < 0.05 based on the Kruskal-Wallis H test. * 0.01< *P* ≤ 0.05; ** 0.001< *P* ≤ 0.01; and ****P* ≤ 0.001. T1, 6:00; T2, 12:00; T3, 18:00; T4, 24:00.


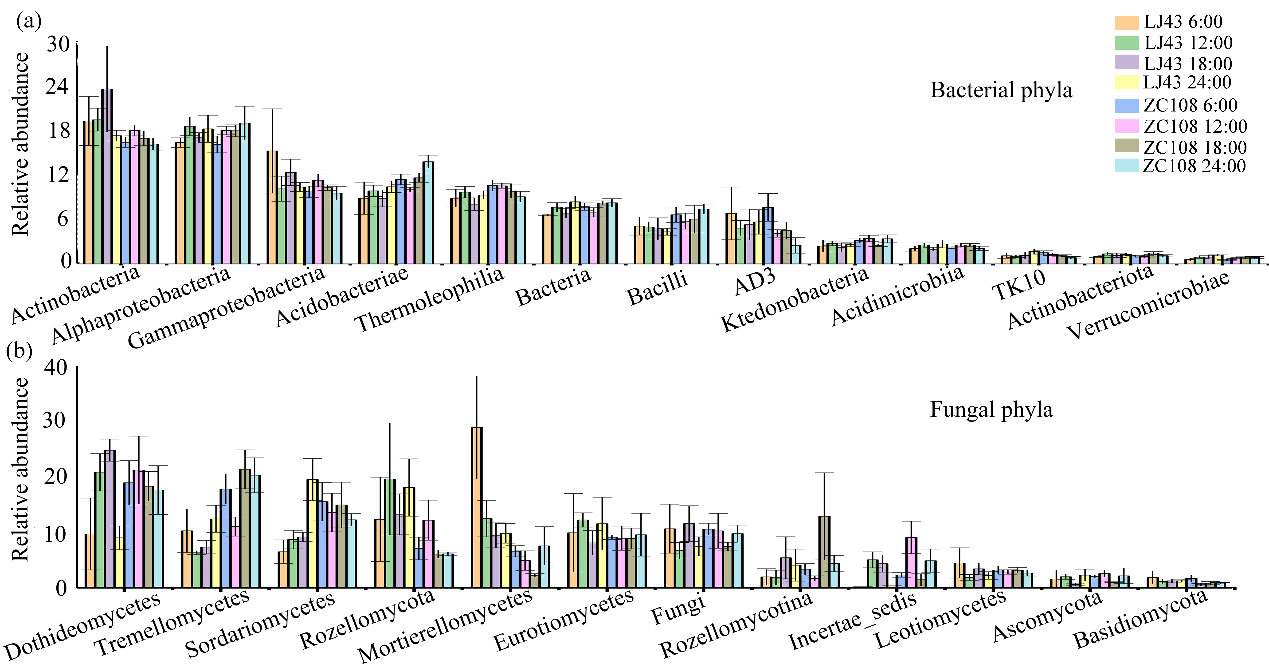


**Figure S3** The multiple comparisons among treatments for the dominant bacterial (a) and fungal (b) taxa were performed with the Kruskal-Wallis H test in the rhizosphere of ‘Longjing43’ (LJ43) and ‘Zhongcha108’ (ZC108).


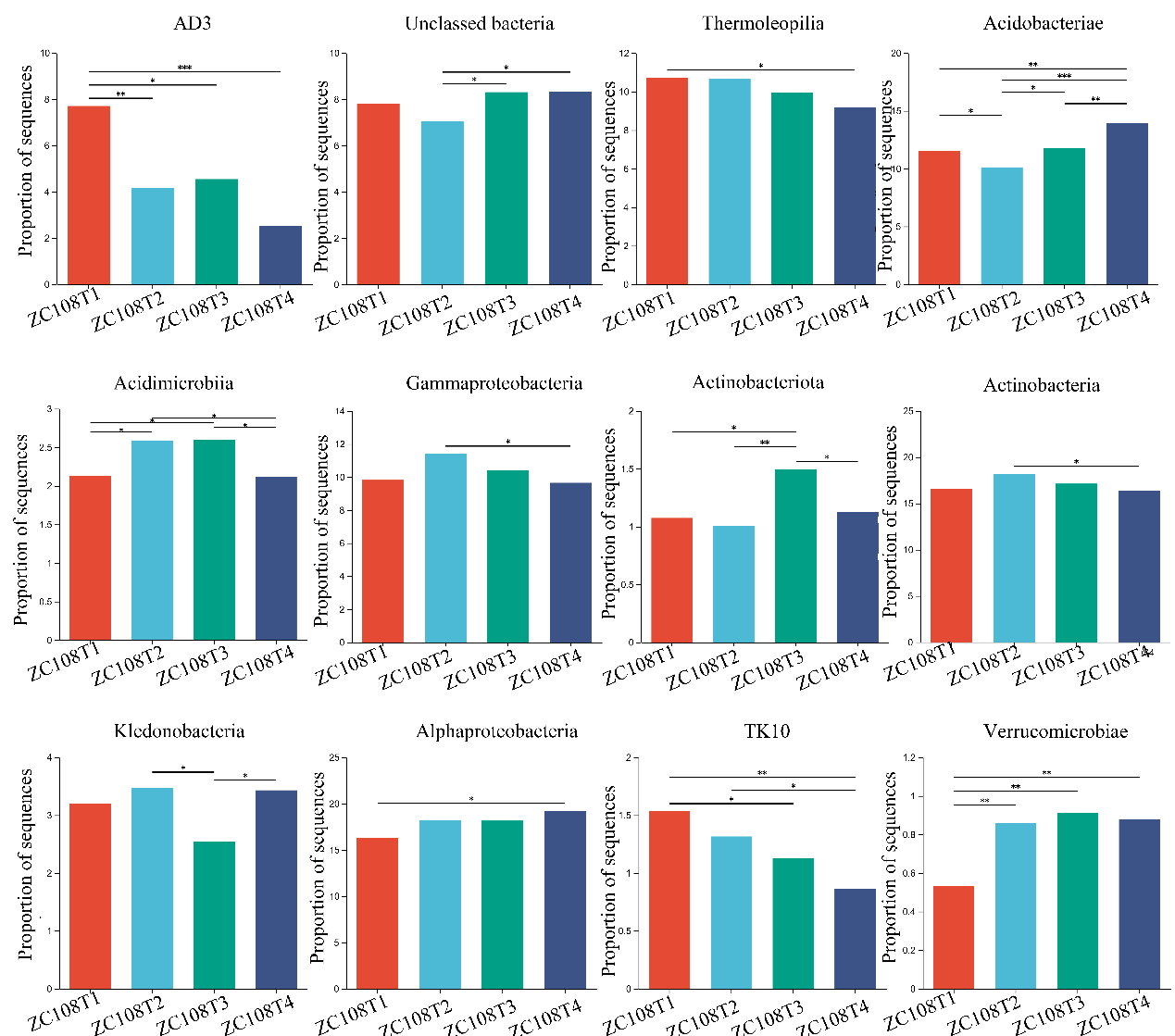


**Figure S4** The dominant bacterial classes in the rhizosphere of tea plants ‘ZhongCha108’ (ZC108) at different times of the day. Different lower-case letters above bars indicate significant differences among treatments at *P* < 0.05 based on the Kruskal-Wallis H test. * 0.01< *P* ≤ 0.05; ** 0.001< *P* ≤ 0.01; and ****P* ≤ 0.001. T1, 6:00; T2, 12:00; T3, 18:00; T4, 24:00.


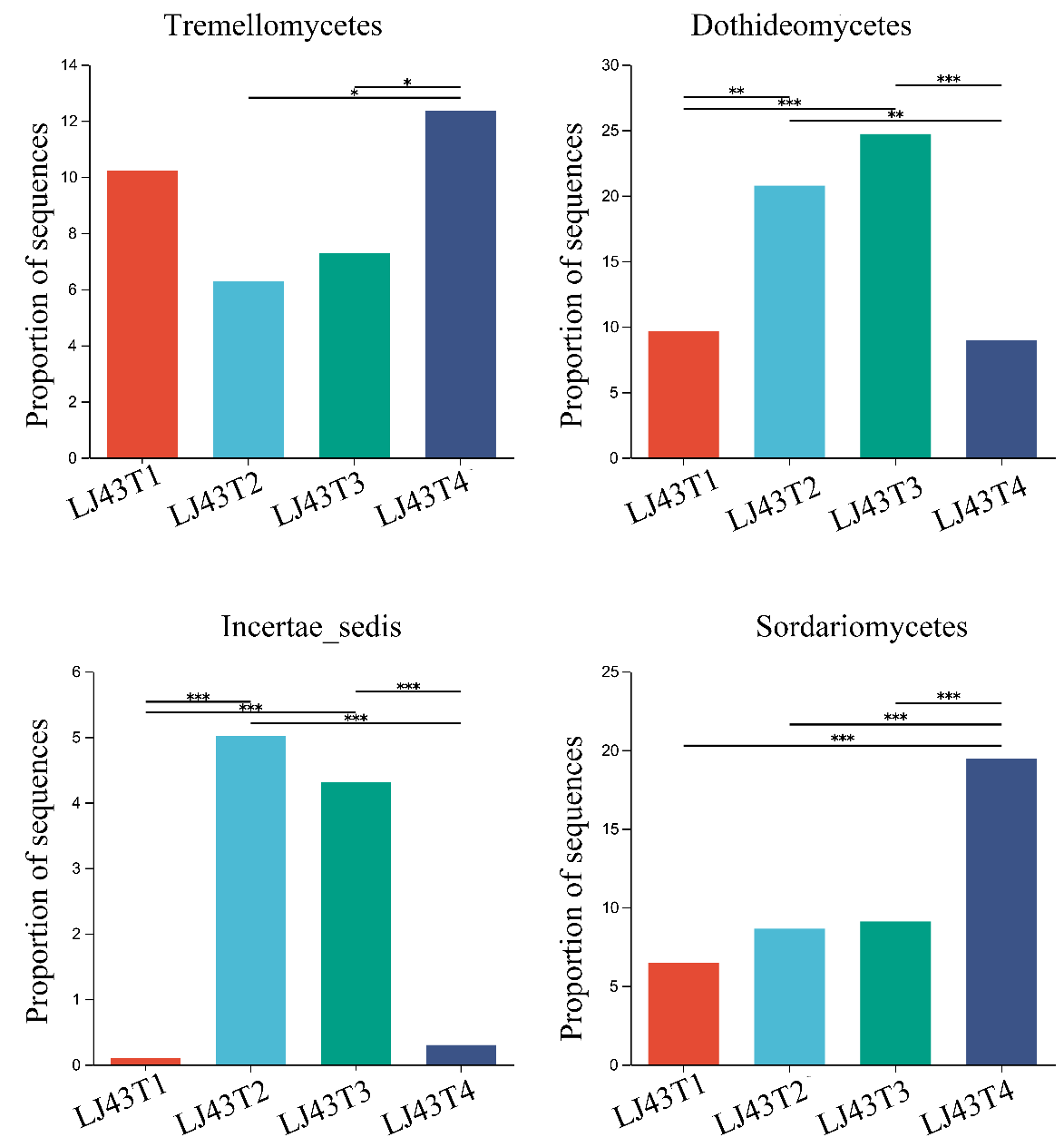


**Figure S5** The dominant fungal classes in the rhizosphere of tea plants ‘Longjing43’ (LJ43) at different times of the day. Different lower-case letters above bars indicate significant differences among treatments at *P* < 0.05 based on the Kruskal-Wallis H test. * 0.01< *P* ≤ 0.05; ** 0.001< *P* ≤ 0.01; and ****P* ≤ 0.001. T1, 6:00; T2, 12:00; T3, 18:00; T4, 24:00.


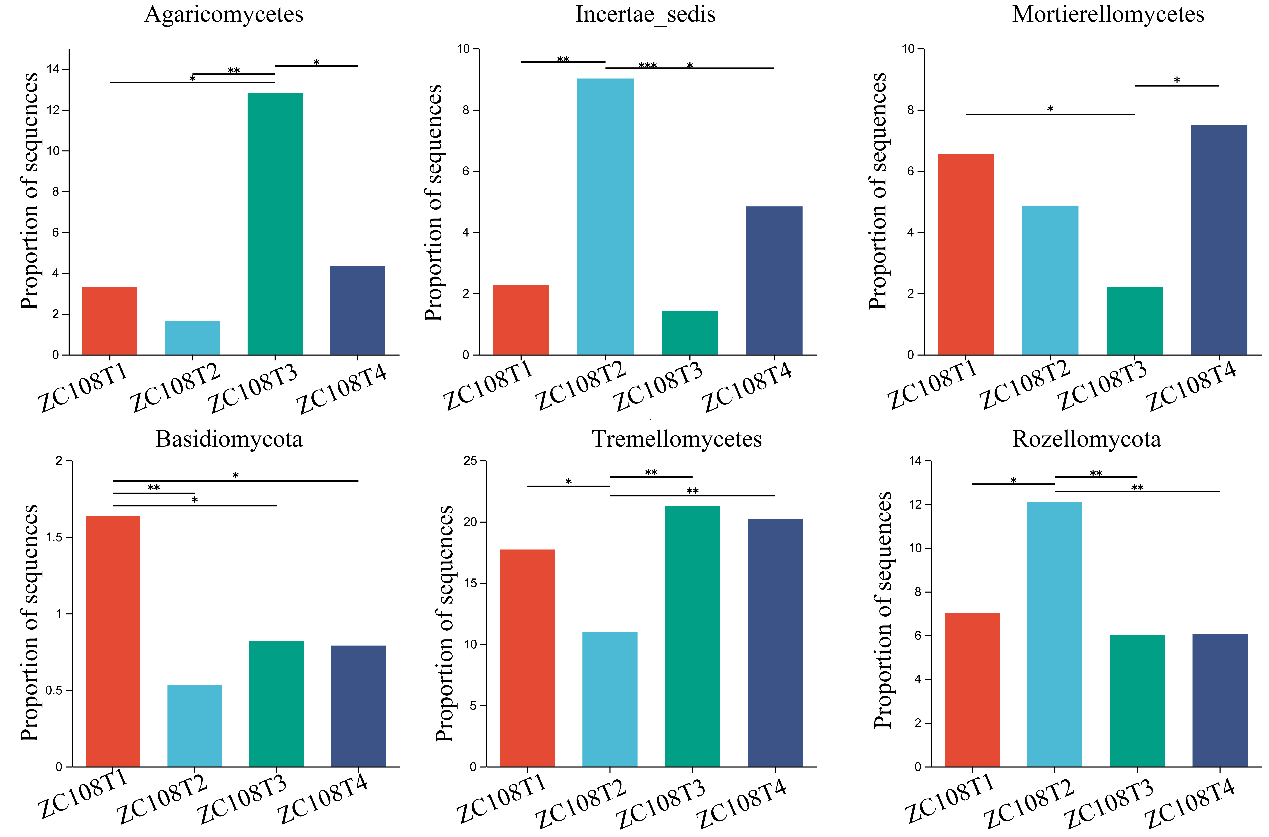


**Figure S6** The dominant fungal classes in the rhizosphere of tea plants ‘Zhongcha108’ (ZC108) at different times of the day. Different lower-case letters above bars indicate significant differences among treatments at *P* < 0.05 based on the Kruskal-Wallis H test. * 0.01< *P* ≤ 0.05; ** 0.001< *P* ≤ 0.01; and ****P* ≤ 0.001. T1, 6:00; T2, 12:00; T3, 18:00; T4, 24:00.


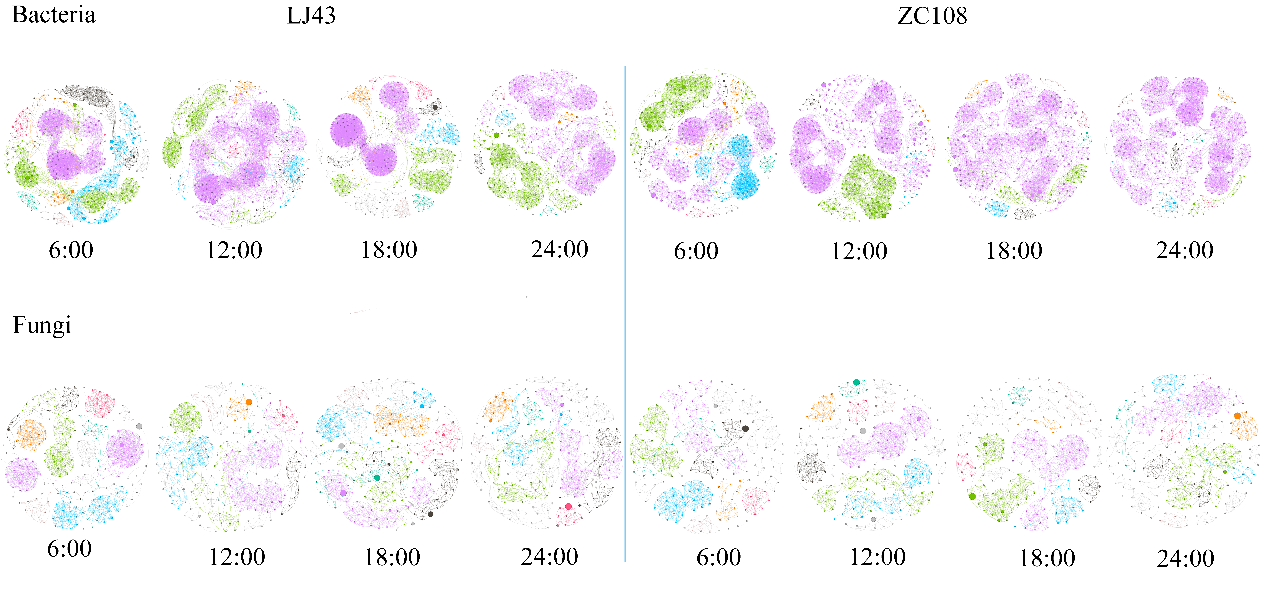


**Figure S7** The network of bacterial and fungal communities in the rhizosphere of ‘Zhongcha108’ (ZC108) and ‘Longjing43’ (LJ43) at time points of 6:00, 12:00, 18:00 and 24:00 of the day. Different node colors represent different cluster modes. The network edges indicate significant correlations (r > 0.75 and *P* < 0.001).


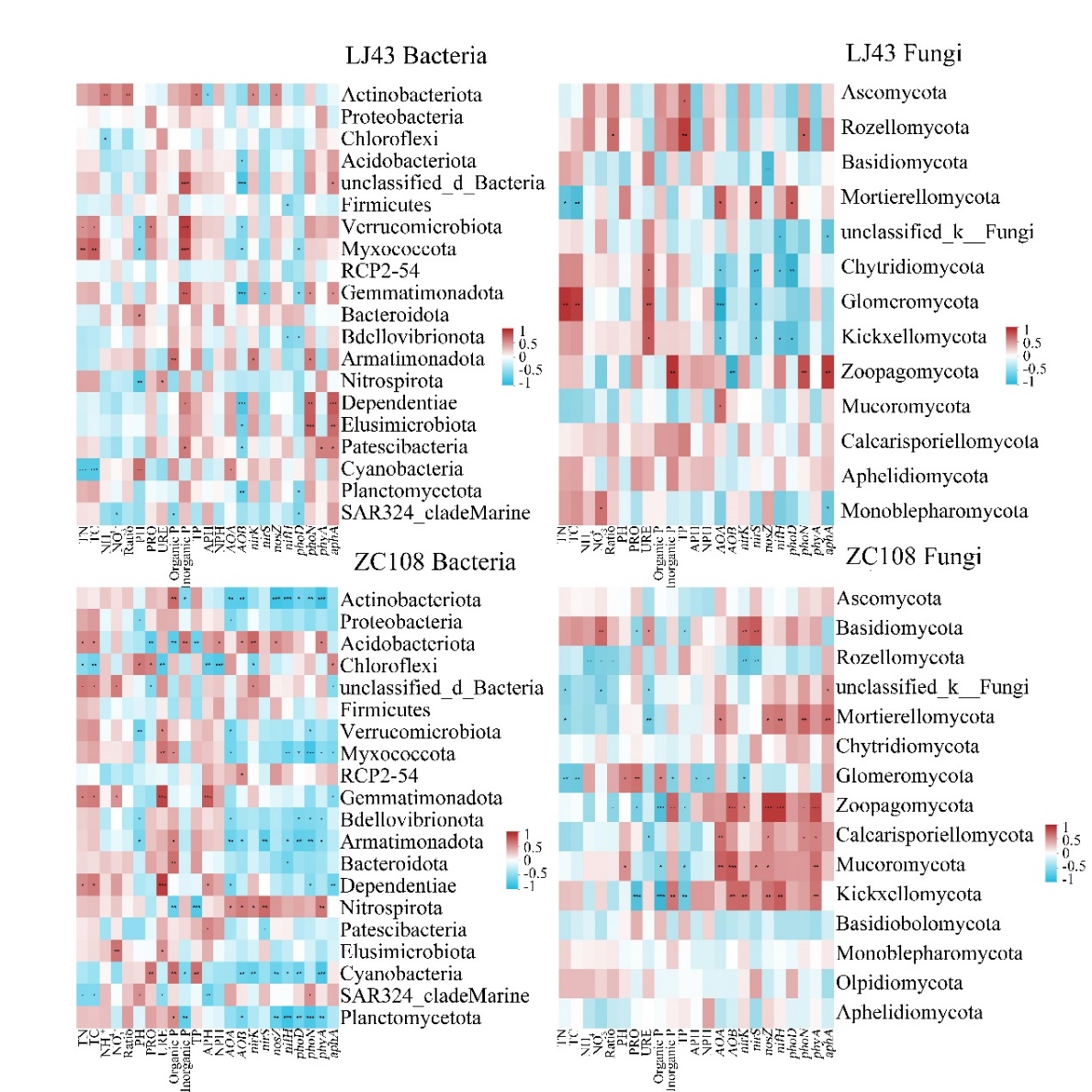


**Figure S8** Heatmaps showing the correlation of soil physiochemical properties and rhizosphere bacterial and fungal community compositions in ‘Longjing 43’ (LJ43) (a) and ‘Zhongcha108’ (ZC108) (b) at time points of 6:00, 12:00, 18:00 and 24:00 of the day. The asterisks represent significant Spearman correlation (0.01 < ^*^*P* < 0.05, 0.01 < ^**^*P* ≤ 0.001; ^***^*P* ≤ 0.001). Red blocks represent positive correlations and blue blocks represent negative correlations. The color strips indicate the size of relative coefficients. TN, total nitrogen, TC, total C, ratio, the ratio of ammonium (NH_4_^+^) to nitrate (NO_3_^－^); PRO, Protease; URE, urease; organic P, organic phosphorus; inorganic P, inorganic phosphorus; TP, total phosphorus; APH, acidic phosphatase; NPH, neutral phosphatase; *AOA* and *AOB*, genes related to nitrification; *nirK*, *nirS* and *nosZ*, genes related to denitrification; *nifH*, gene related to nitrogen fixation; *phoD* and *phyA*, genes related to alkaline phosphatase activity; *aphA* and *phoN*, genes related to acidic phosphatase activity.

**Table S1** Soil properties including soil pH, total carbon (TC, g kg^-1^), total nitrogen (TN, g kg^-1^), nitrate (NO_3_^−^, mg kg^-1^), ammonium (NH_4_^+^, mg kg^-1^), total phosphorus (TP, mg kg^-1^) and organic P (mg kg^-1^) in the rhizosphere of two tea species‘Longjing43’ (LJ43) and ‘Zhongcha108’ (ZC108) at different times of the day. Different lower-case letters indicate significant differences among treatments at *P* < 0.05 based on ANOVA followed by Duncan’s tests. T1, 6:00; T2, 12:00; T3, 18:00; T4, 24:00.

| Tea species | pH | TN | TC | TP | NO_3_^−^ | NH_4_^+^ | Organic P |
| --- | --- | --- | --- | --- | --- | --- | --- |
| LJ43 |  |  |  |  |  |  |  |
| T1 | 3.70±0.05bc | 1.32±0.05e | 13.6±0.44d | 8.67±1.26b | 22.3±2.16b | 13.2±1.57d | 10.2±1.40bc |
| T2 | 3.71±0.01b | 1.33±0.05e | 13.9±0.49d | 10.24±0.34a | 24.6±1.32ab | 22.1±2.17b | 12.8±0.50a |
| T3 | 3.67±0.01bcd | 1.89±0.09a | 19.9±0.44a | 9.29±0.14ab | 25.6±0.82a | 22.9±2.07b | 11.8±0.36ab |
| T4 | 3.66±0.01d | 1.80±0.09a | 19.2±0.89a | 8.40±0.43b | 19.1±2.56c | 13.2±2.63d | 11.5±0.51ab |
| ZC108 |  |  |  |  |  |  |  |
| T1 | 3.76±0.04a | 1.38±0.04de | 14.3±0.25d | 6.22±0.51c | 19.1±2.09c | 21.6±1.32b | 10.4±1.07bc |
| T2 | 3.65±0.02d | 1.44±0.04cd | 15.2±0.24c | 9.04±0.68b | 17.5±1.37c | 17.9±2.33c | 12.5±1.08a |
| T3 | 3.67±0.01cd | 1.57±0.09b | 16.6±0.85b | 6.31±1.10c | 22.2±0.62b | 29.4±1.75a | 9.28±1.99c |
| T4 | 3.64±0.01d | 1.50±0.05bc | 16.1±0.22b | 4.70±0.45d | 18.9±1.97c | 13.5±0.61d | 8.99±1.09c |

**Table S2** Primer pairs for the genes related to soil nitrogen and phosphorus cycling.

| Gene name | Primer name | Forward primer | Reverse primer | Size | References |
| --- | --- | --- | --- | --- | --- |
| *nifH* | ploF_ploR | TGCGAYCCSAARGCBGACTC | ATSGCCATCATYTCRCCGGA | 450 | Tu et al. 2015 |
| *nirK* | nirK-C2F_nirK-C2R | TGCACATCGCCAACGGNATGTWYGG | GGCGCGGAAGATGSHRTGRTCNAC | 448 | Wei et al. 2015 |
| *nirS* | nirS-C1F_nirS-C1R | ATCGTCAACGTCAARGARACVGG | TTCGGGTGCGTCTTSABGAASAG | 500 | Wei et al. 2015 |
| *AOB* | CTO189f_CTO654R | GGAGRAAAGCAGGGGATCG | CTAGCYTTGTAGTTTCAAACGC | 466 | Kowalchuk et al. 1997 |
| *AOA* | CHEND-arch-amoA-23F_CHEND-arch-amoA-616R | ATGGTCTGGCTWAGACG | GCCATCCATCTGTATGTCCA | 629 | Sahan and Muyzer, 2008 |
| *nosZ* | MQX-nosZ-1F_MQX-nosZ-1622R | CGYTGTTCMTCGACAGCCAG | CGSACCTTSTTGCCSTYGCG | 500 | Henry et al. 2006 |
| *phoD* | phoD | ACCTGGGACGATCAYGARGTNGA | GCCACATAACCRTCCCANTKRTC | 450 | Rose et al. 2003 |
| *phoN* | phoN-Sf | GGAAGAACGGCTCCTACCCIWSNGGNCA | CACGTCGGACTGCCAGTGIDMIYYRCA | 208 | Rossolini et al. (1998 |
| *aphA* | aphA-1a | AACGTCTTGCTCGAGGCCGCG | GGCAAGATCCTGGTATCGGTCTGC | 669 | Sandvang and Aarestrup, 2000 |
| *phoA* | phoA | ATCTTCATCCACCCNGAYGGNAC | AAGTTGTCGGTGCCYTCYTCYTC | 800 | Rose et al. 2003 |

**Table S3** Soil phosphorus (P) fractions (mg/kg): Resin-P, NaHCO_3_-P_t_, NaHCO_3_-P_i_, NaOH-P_t_, HCl-P, Residual P and organic P in the rhizosphere of two tea species ‘Longjing43’ (LJ43) and ‘Zhongcha108’ (ZC108) at different times of the day. Different lower-case letters indicate significant differences among treatments at *P* < 0.05 based on ANOVA followed by Duncan’s tests. T1, 6:00; T2, 12:00; T3, 18:00; T4, 24:00.

| Tea species | Resin-P | NaHCO_3_-P_t_ | NaOH-P_t_ | HCl-P | Residual P |
| --- | --- | --- | --- | --- | --- |
| LJ43 |  |  |  |  |  |
| T1 | 6.27±0.16f | 18.42±1.75d | 54.24±2.36ab | 124.37±8.67de | 776.92±137.74bc |
| T2 | 9.98±0.37e | 19.38±0.72cd | 58.12±0.66a | 133.67±10.62d | 1008.43±57.79a |
| T3 | 11.01±0.41e | 19.26±0.46cd | 56.63±3.19ab | 115.41±2.07e | 931.57±41.72ab |
| T4 | 12.34±0.59d | 19.47±0.81cd | 54.44±2.87ab | 136.77±4.95d | 885.69±57.02ab |
| ZC108 |  |  |  |  |  |
| T1 | 20.85±0.58a | 22.33±1.70a | 55.81±3.57ab | 189.82±14.11b | 709.12±126.67cd |
| T2 | 14.17±1.48c | 19.76±1.91bcd | 53.66±2.35b | 171.56±2.39c | 949.93±107.01ab |
| T3 | 14.17±0.85c | 20.65±1.14abc | 57.89±2.90ab | 199.81±17.68b | 586.95±212.07de |
| T4 | 18.61±0.56b | 21.49±0.88ab | 57.58±2.31ab | 248.04±16.99a | 506.38±17.58e |

**References**

Tu Q, Zhou X, He Z, Xue K, Wu L, Reich P, et al. (2016). The diversity and co-occurrence patterns of N2-fixing communities in a CO_2_-enriched grassland ecosystem. Microb Ecol 71: 604-615.

Kowalchuk GA, Stephen JR, De Boer W, Prosser JI, Embley TM, Woldendorp JW. (1997) Analysis of ammonia-oxidizing bacteria of the beta subdivision of the class Proteobacteria in coastal sand dunes by denaturing gradient gel electrophoresis and sequencing of PCR-amplified 16S ribosomal DNA fragments. Appl Environ Microbiol 63: 1489-1497.

Sahan E, Muyzer G. (2008). Diversity and spatio-temporal distribution of ammonia-oxidizing archaea and bacteria in sediments of the Westerschelde estuary. FEMS Microbiol Ecol 64: 175-186.

Henry S, Bru D, Stres B, Hallet S, Philippot L. (2006). Quantitative detection of the nosZ gene, encoding nitrous oxide reductase, and comparison of the abundances of 16S rRNA, *narG*, *nirK*, and *nosZ* genes in soils. Appl Environ Microb 72: 5181-5189.

Wei W, Isobe K, Nishizawa T, Zhu L, Shiratori Y, Ohte N. et al. (2015) Higher diversity and abundance of denitrifying microorganisms in environments than considered previously. ISME J 9: 1954-1965.

Sandvang D, Aarestrup FM. (2000) Characterization of aminoglycoside resistance genes and class 1 integrons in porcine and bovine gentamicin-resistant Escherichia coli. Microbial Drug Resistance 6: 19-27.

Rose TM, Henikoff JG, Henikoff S. (2003) CODEHOP (COnsensus-DEgenerate hybrid oligonucleotide primer) PCR primer design. Nucleic Acids Res 31: 3763-3766.

Rossolini GM, Schippa S, Riccio ML, Berlutti F, Macaskie LE, Thaller MC. (1998) Bacterial nonspecific acid phosphohydrolases: physiology, evolution and use as tools in microbial biotechnology. Cell Mol Life Sci 54: 833-850.
